# Supplementary material for: α-amino trimethylation of CENP-A by NRMT is required for full recruitment of the centromere
Source: Nat Commun. 2017 Mar 7;8:14678. doi: 10.1038/ncomms14678 (PMC5343448; doi:10.1038/ncomms14678)
Supplement: Supplementary Information — Supplementary Figures, Supplementary Methods, and Supplementary References [file ncomms14678-s1.pdf]

Supplementary Fig.1

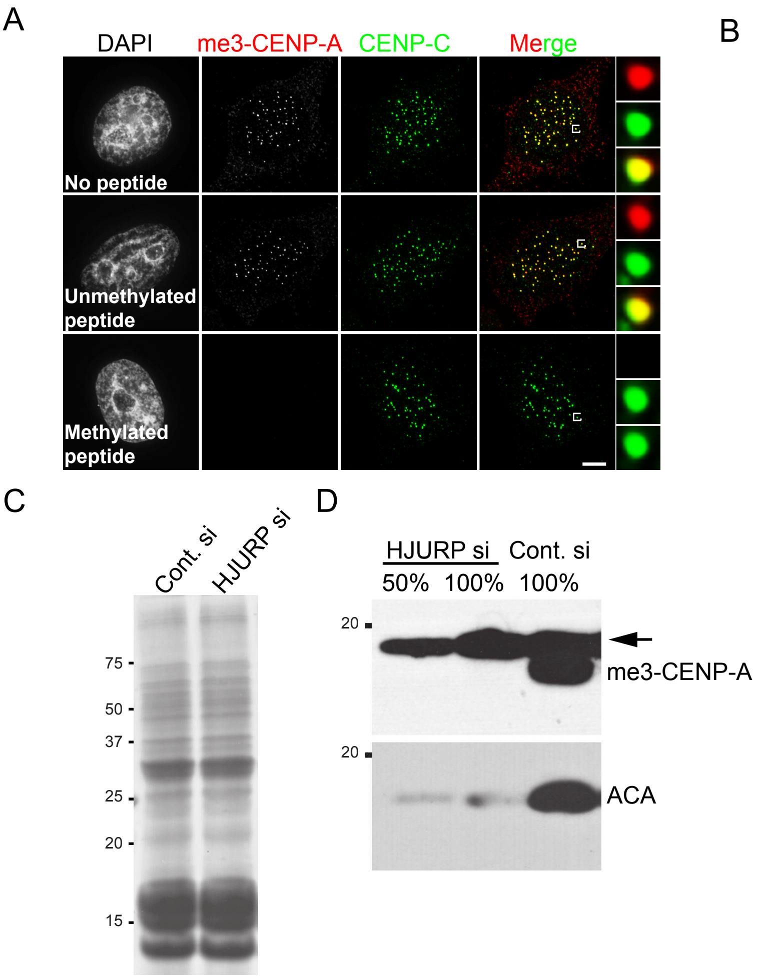

Supplementary Figure 1. Anti-me3-CENP-A antibody specifically detects methylated CENP-A at the centromere (A) Peptide blocking experiment showing anti me3-CENP-A antibody specifically blocked by methylated peptides. (B) CENP-A knockdown followed by Immunofluorescence analysis in HEK cells using me3-CENP-A antibody showing complete lack of centromere staining. (C) Acid extracted histones after siRNA mediated HJURP knockdown. (D) me3-CENP-A antibody detects CENP-A in the control but not in the HJURP knockdown samples. Arrow indicates non-specific band. Scale bar, 10 μm.

Supplementary Fig.2

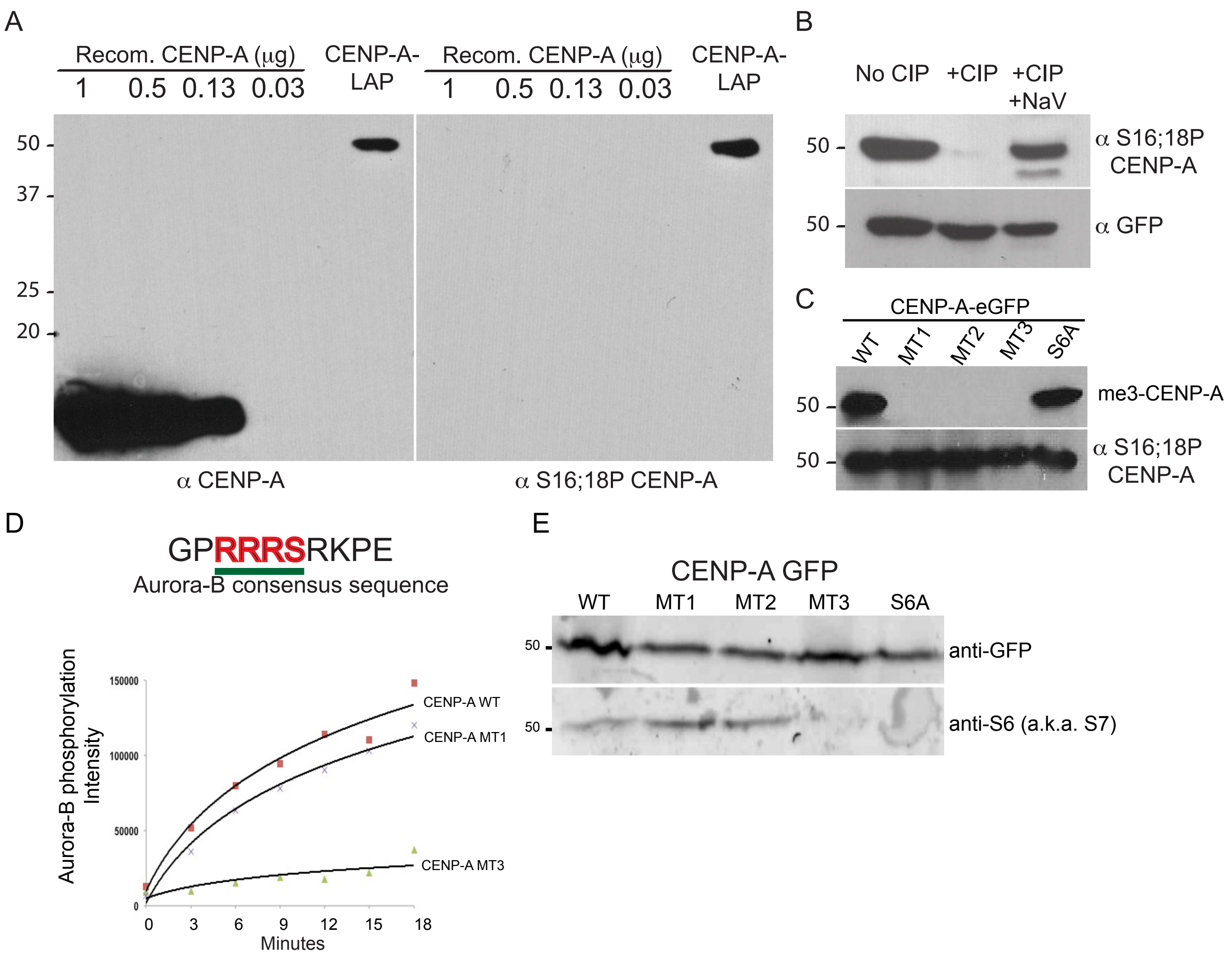

Supplementary Figure 2. Phosphorylation status of CENP-A amino tail in CENP-A methylation mutants (A) Specificity of CENP-A S16;18 phospho antibody. The western blot analysis of purified CENP-A shows that the antibody did not detect purified CENP-A but CENP-A-eGFP in the cell lysate. (B) Treatment of the lysate with phosphatase abolished the detection of the CENP-A by the antibody and is rescued by treating phosphatase along with sodium vanadate. (C) Western blot showing methylation mutants MT1, 2 and 3 phosphorylated at S16:18. (D) Aurora B kinase assay showing MT3 is not phosphorylated. CENP-A Aurora B consensus sequence is shown. (E) CENP-A wild type and mutants were analyzed by western blotting for S6 phosphorylation and similar to kinase assay, MT3 was not phosphorylated.

Supplementary Fig.3

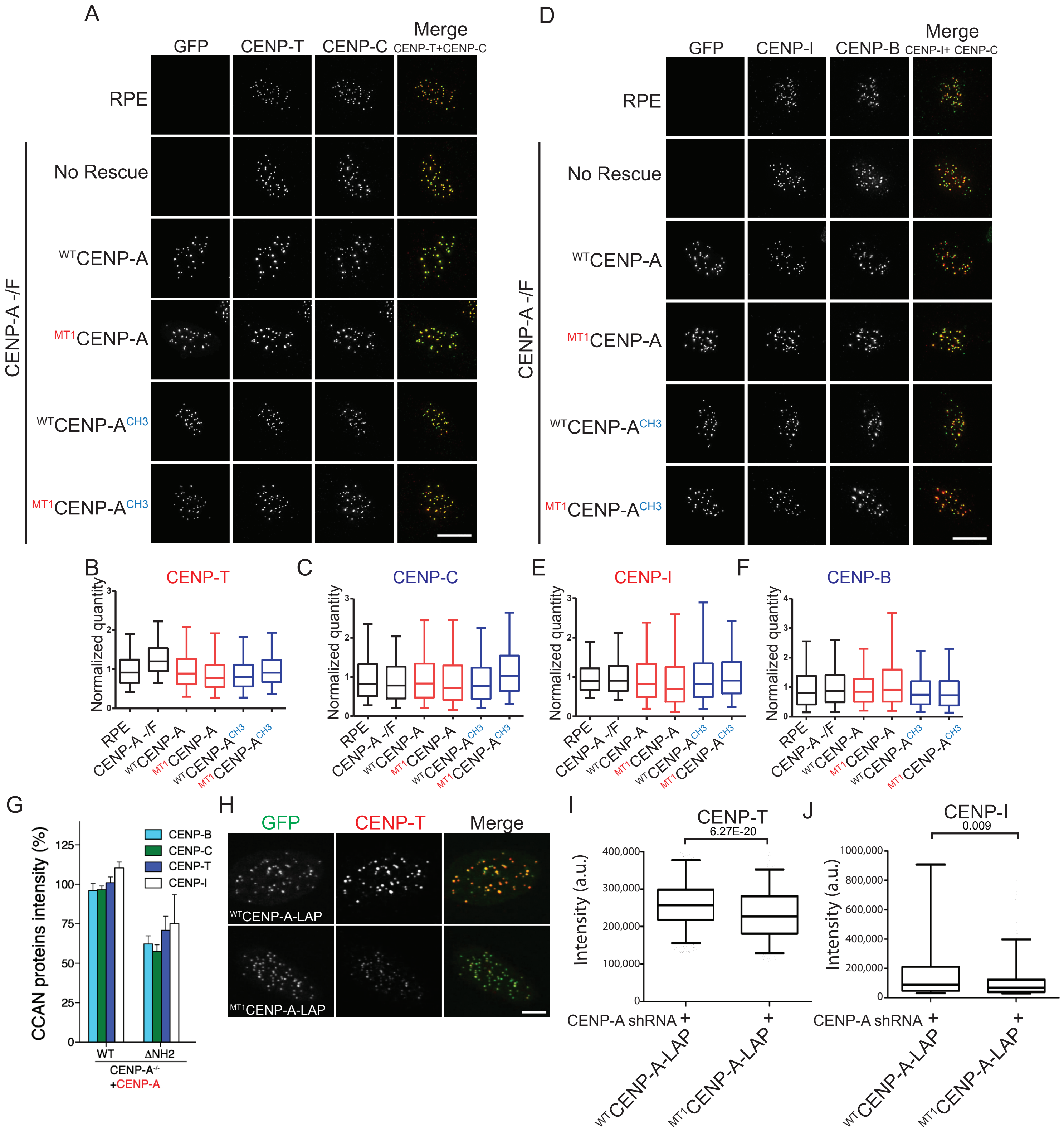

Supplementary Figure 3. CCAN components did not change drastically in cells expressing wild type or mutant exogenous CENP-A in the presence of endogenous CENP-A (A) Representative images of the cells stained for CENP-T and CENP-C. (B&C) Quantitation of CENP-T and CENP-C respectively at the centromere in cells expressing endogenous CENP-A. (D) Representative images of the cells stained for CENP-I and CENP-B. (E&F) Quantitation of CENP-I and CENP-B respectively at the centromere. More than 30 cells in two biological replicates used for the quantitation. Box-and-whisker plots. Central lines, medians; whiskers, range 5-95 percentile; outliers not shown, Scale bar, 10 μm. (G) Localization of CENP-T, CENP-I, CENP-C and CENP-B at the centromere after replaced with CENP-A N-terminal tail deletion mutant. Error bars indicate standard deviation. (H) CENP-T localization to the centromere reduced in HeLa-TRex cells replaced with mutant CENP-A. The endogenous CENP-A is suppressed by CENP-A 3'UTR shRNA. (I&J) Quantitation of the CENP-T and CENP-I level after knockdown and replacement of the endogenous CENP-A using Velocity 5.5. Box-and-whisker plots. Central lines, medians; whiskers, range 5-95 percentile; outliers not shown. p-values determined by t-test.

Supplementary Fig.4

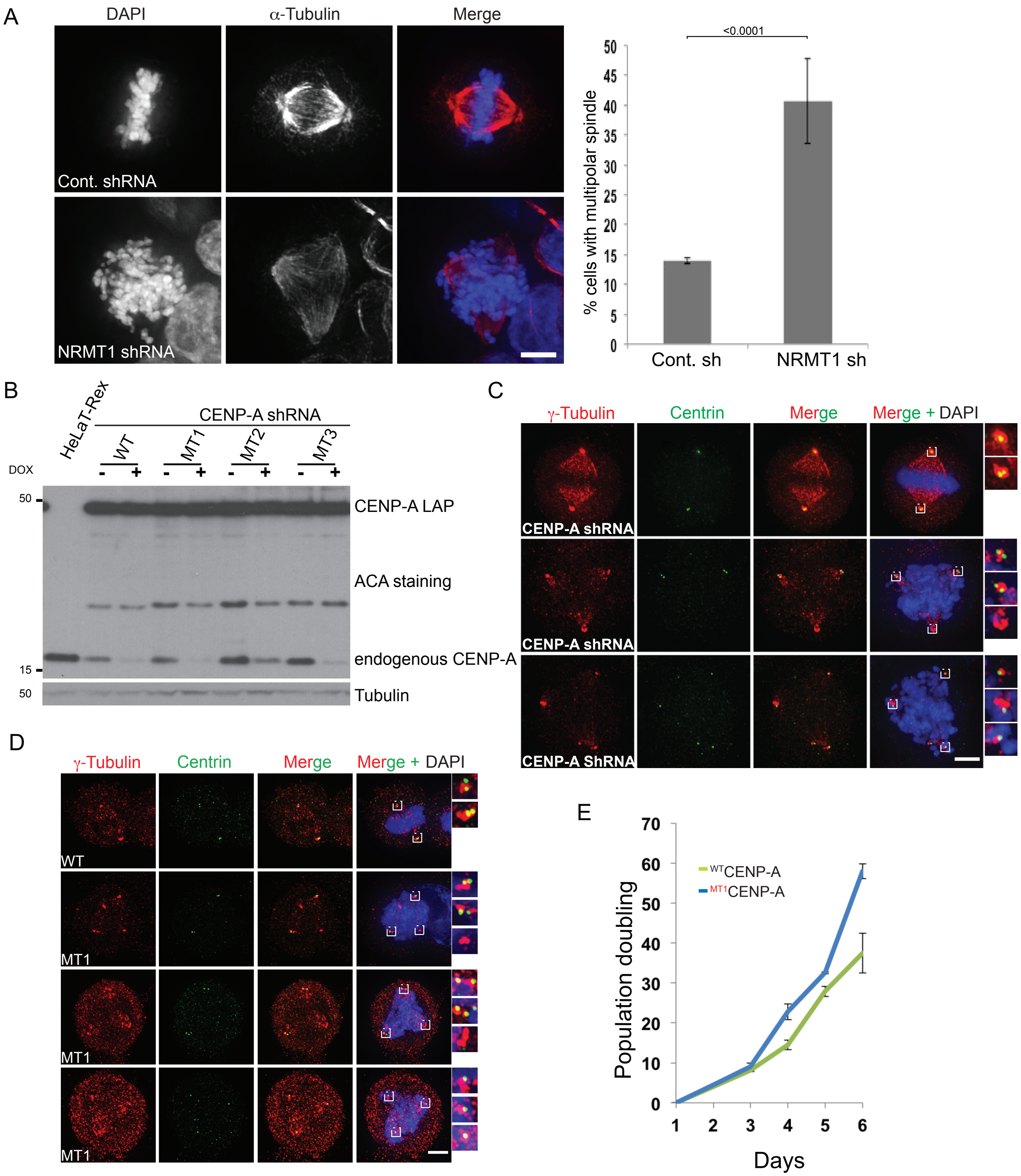

Supplementary Figure 4. NRMT1 knockdown and CENP-A methylation mutant result in the same phenotype (A) NRMT1 knockdown causes multipolar phenotype. Control vs NRMT1 shRNA given in the graph. Mean and standard deviation shown from three different experiments. χ^2 test was used. (B) Endogenous CENP-A knockdown efficiency in CENP-A knockdown replacement experiments. (C) Multipolarity caused by centriole disengagement and/or pericentriolar matrix splitting in CENP-A knockdown experiments and knockdown and replacement experiments. (D) Images of different types of centrosomes with or without centrioles seen after CENP-A knockdown are shown. Scale bar, 10 µm. (E) The population doubling of the HCT116 p53-/- cells replaced with ^{WT}CENP-A or ^{MT1}CENP-A. Endogenous CENP-A was knockdown using 3'UTR shRNA. Experiment was conducted in triplicate. Error bars indicate standard deviation.

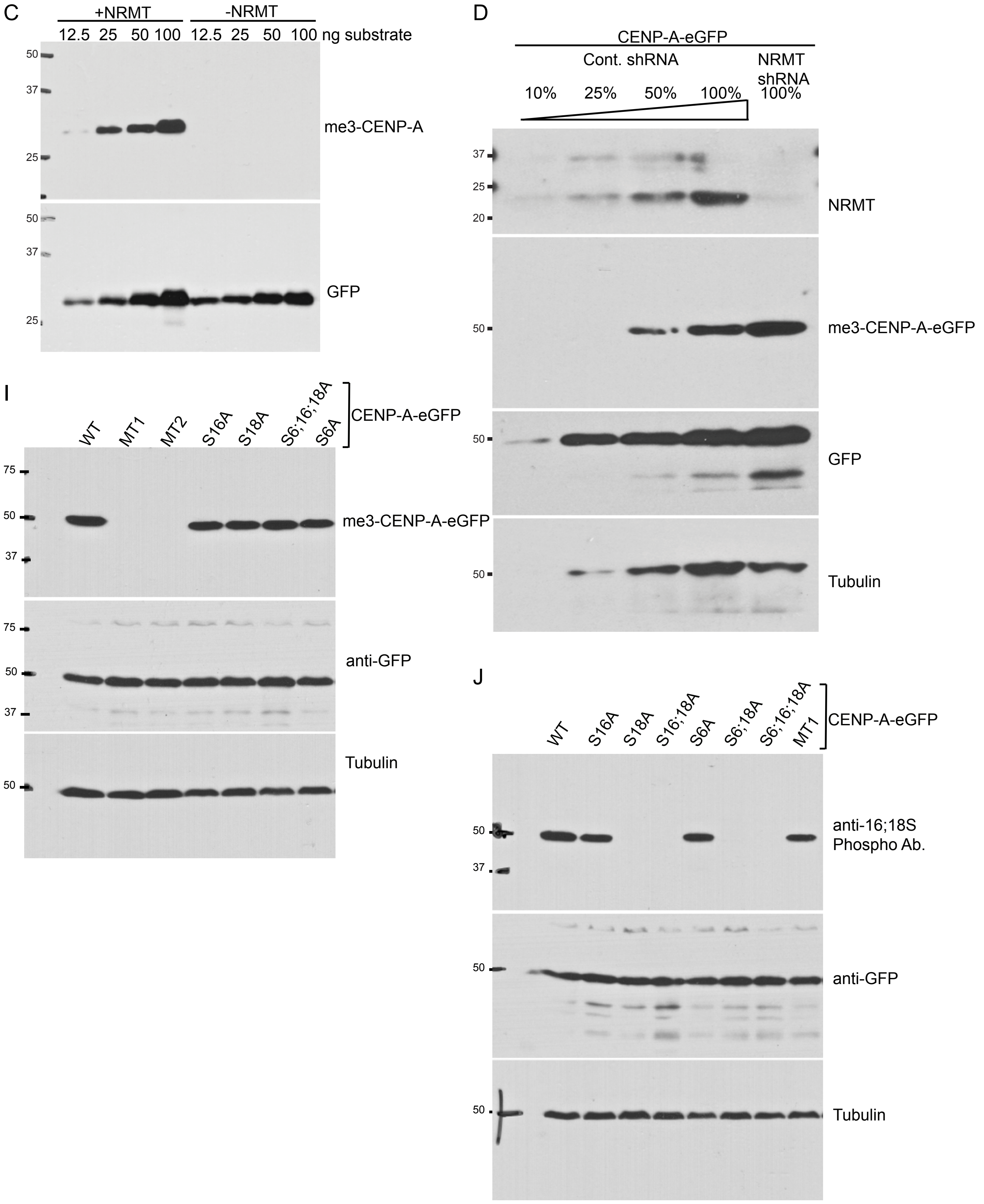

Supplementary Figure 5. Uncropped blots from figure 1.

Figure 4 D

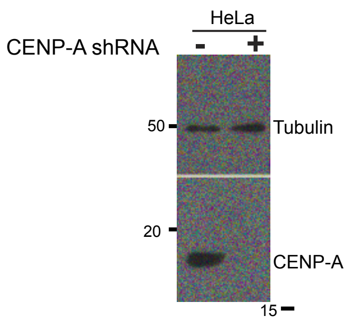

Figure 5 B

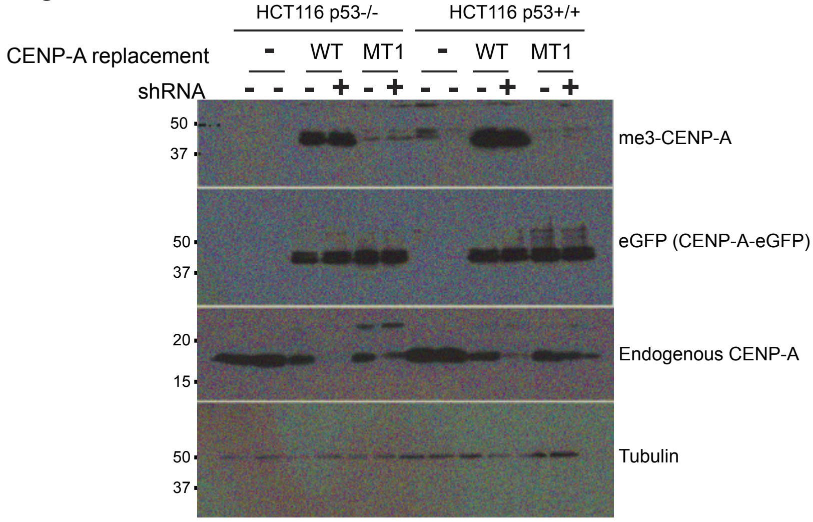

Figure 6 B

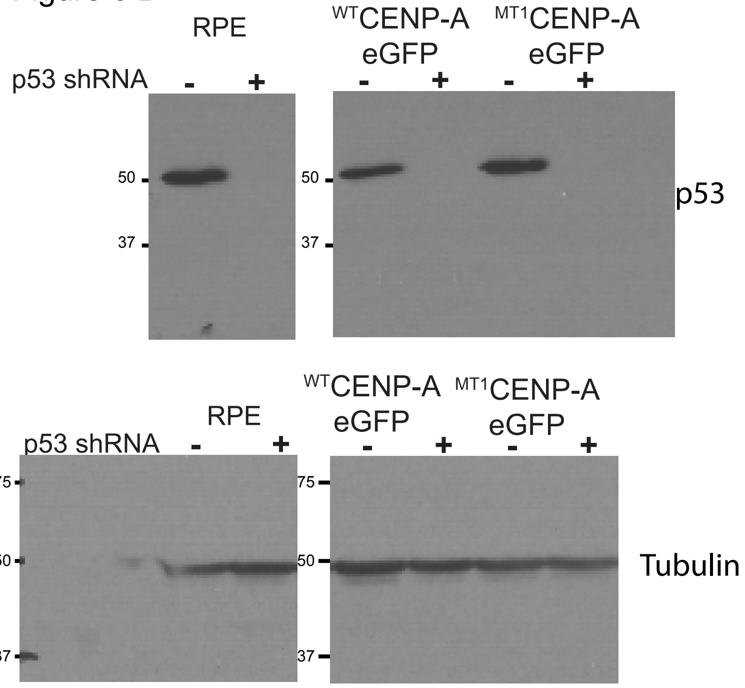

Figure 6 E

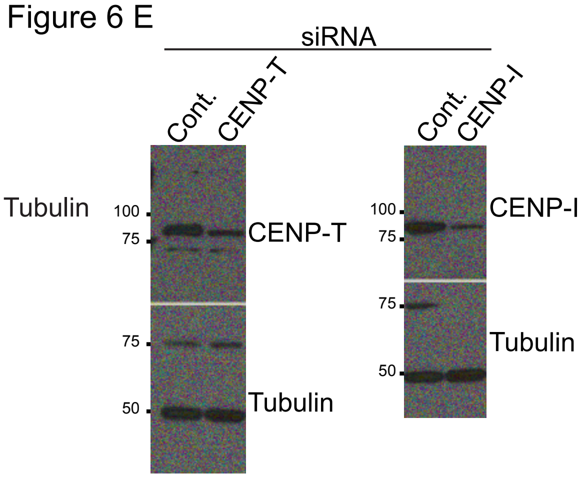

Supplementary Figure 6. Uncropped blots from figures 4, 5 and 6 as indicated.

Supplementary Methods

Acid extraction of histones: The histones were acid extracted from $2X10^7$ HeLa cells after HJURP or control siRNA knocked down for 72 hours [1, 2]. Briefly, the cells were treated with hypotonic buffer for 30 minutes on a rotator. Pelleted the nuclei and added 400μl of 0.4N H_2SO_4. Histones were extracted for 30 minutes and then TCA precipitated from the supernatant.

Peptide blocking: The diluted anti-me3 CEMP-A antibody pre-incubated with 10 times peptides (methylated or un-methylated peptides) for 30 minutes. The antibody then centrifuged at 12,000xg for 5 minutes and used for immunofluorescence as described before.

Supplementary References

1. Shechter, D., Dormann, H.L., Allis, C.D. & Hake, S.B. Extraction, purification and analysis of histones. *Nature protocols* **2**, 1445-1457 (2007).
2. Foltz, D.R. *et al.* Centromere-specific assembly of CENP-a nucleosomes is mediated by HJURP. *Cell* **137**, 472-484 (2009).
